# Characterization and Fine Mapping of *qRPR1-3* and *qRPR3-1*, Two Major QTLs for Rind Penetrometer Resistance in Maize

**DOI:** 10.3389/fpls.2022.944539

**Published:** 2022-07-19

**Authors:** Xinwei Hou, Senan Cheng, Shukai Wang, Ting Yu, Yancui Wang, Pingping Xu, Xitong Xu, Qi Zhou, Xuetong Hou, Guobin Zhang, Cuixia Chen

**Affiliations:** State Key Laboratory of Crop Biology, College of Agronomy, Shandong Agricultural University, Tai'an, China

**Keywords:** maize, lodging resistance, QTL, RPR, fine mapping

## Abstract

Stalk strength is one of the most important traits in maize, which affects stalk lodging resistance and, consequently, maize harvestable yield. Rind penetrometer resistance (RPR) as an effective and reliable measurement for evaluating maize stalk strength is positively correlated with stalk lodging resistance. In this study, one F_2_ and three F_2:3_ populations derived from the cross of inbred lines 3705I (the low RPR line) and LH277 (the high RPR line) were constructed for mapping quantitative trait loci (QTL), conferring RPR in maize. Fourteen RPR QTLs were identified in four environments and explained the phenotypic variation of RPR from 4.14 to 15.89%. By using a sequential fine-mapping strategy based on the progeny test, two major QTLs, *qRPR1-3* and *qRPR3-1*, were narrowed down to 4-Mb and 550-kb genomic interval, respectively. The quantitative real-time PCR (qRT-PCR) assay was adopted to identify 12 candidate genes responsible for QTL *qRPR3-1*. These findings should facilitate the identification of the polymorphism loci underlying QTL *qRPR3-1* and molecular breeding for RPR in maize.

## Introduction

Maize has become one of the most important food crops because it can be used as staple grain, fodder, and industrial raw material (Hu et al., 2012). Notably, stalk lodging reduced annual maize yields by 5–20% (Flint-Garcia et al., 2003b). Besides restricting maize yield and quality, stalk lodging is not conductive to mechanical harvest, and increases labor cost consequently (Zhang et al., 2020). Therefore, improving stalk-lodging resistance has been a key objective for maize breeding programs.

Stalk lodging is closely related to maize stalk strength, which was controlled by the morphology and cell wall structure components of stalk (Zuber et al., 1980; Robertson et al., 2017). So far, there have been several testing methods for evaluating stalk strength, including mechanical methods, chemical methods, and morphological measurement. Among them, the mechanical methods can evaluate the stalk strength of maize with large population in a short period, which is minimally required for the phenotypic measurement for fine mapping. Stalk crushing strength (SCS) (Zuber and Grogan, 1961), stalk bending strength (SBS) (Sekhon et al., 2020), and rind penetrometer resistance (RPR) (Dudley, 1994; Liu et al., 2020) are three typical mechanical methods in the field and positively correlated with stalk lodging resistance. Among them, RPR overcomes the drawbacks of SCS and SBS, including increasing labor cost and destructing maize stalk (Robertson et al., 2017), and has been widely applied to crop genetic breeding (Albrecht and Dudley, 1987; Abedon et al., 1999; Hu et al., 2000; Feng et al., 2009).

According to several genetic studies, RPR is regulated by a large number of QTL. Thirty-five individual QTLs and 11 pairs of epistatic interactions for RPR were detected from the four F_2:3_ maize populations. The major QTL accounted for > 15% of the total phenotypic variation of RPR (Flint-Garcia et al., 2003a). By constructing a recombinant inbred line (RIL) population crossed with the inbred lines Ce03005 and B73, nine additive-effect QTLs and one pair of epistatic interaction were identified and each QTL could explain the phenotypic variation of RPR from 1.15% (bin, 6.01) to 12.43% (bin, 3.06) (Hu et al., 2012). Across nested association mapping (NAM) and intermated B73 × Mo17 (IBM) families with more polymorphic loci and bigger mapping population, 18 QTLs and 141 significant GWAS associations for RPR were identified (Peiffer et al., 2013). Approximately, 3,072 SNPs in the GoldenGate maize SNP assay that could increase QTL resolution to make that seven QTLs for RPR were identified in the two RIL populations, the parents of which were H127R and Chang7-2 lines, and B73 and By804 lines, respectively. These QTLs could account for the RPR phenotypic variation from 4.4 to 18.9% (Li et al., 2014). Recently, *stiff1*, which encodes an F-box domain protein, has been identified for regulating stalk strength in maize. There is a 27.2 kb transposable element insertion in the promoter of *stiff1*, leading to increase in the content of cellulose and lignin in maize stalk and, consequently, higher stalk strength (Zhang et al., 2020). The identification of *stiff1* paves a new way for the breeding program of maize stalk strength. However, the QTLs of stalk strength are still harder to be identified because of the complication of maize genome.

In this study, two inbred lines with great difference in RPR were chosen to construct F_2_ and F_2:3_ families for discovering more loci, conferring higher RPR to maize. By using a sequential fine-mapping strategy based on the progeny test (Ma et al., 2017), the physical region of two major QTLs, *qPRP1-3* and *qPRP3-1*, was narrowed down to 4 MB and 550 kb. A qRT-PCR assay was adopted to identify the candidate gene responsible for *qPRP3-1*. These results provide a basis for facilitating molecular breeding of stalk strength and identifying causal gene underlying *qPRP3-1*.

## Materials and Methods

### Field Experiments and RPR Measurement

The F_2_ and F_2:3_ genetic population consisting of 214 individuals/families was constructed by single-seed descent from the cross between 3705I and LH277. In the summer of 2015, F_2_ population was planted at the experimental station of Shandong Agricultural University, Tai'an, China (36.18°N, 117.13°E). In the winter of 2015, F_2:3_ population obtained from self-pollinated F_2_ population was planted at the experimental station of Sanya, Hainan, China (18.15°N, 109.30°E). In the summer and winter of 2016, another two replications of F_2:3_ populations were planted at Tai'an and Sanya, respectively. Each line was grown in a single 2.8-m row; rows were 0.6 m apart, and planting density was 83,000 plants/ha. At the tasseling stage at the average level of each population, the RPR of five randomly selected plants in each row was measured against the three flat sides of the third internodes below the primary ear by using electronic penetrometer (ZTS-50N, IMADA Company, Japan; Supplementary Figure 1).

### Phenotypic Data Analysis

Four sets of phenotypic data were obtained for the F_2_ and F_2:3_ populations, corresponding to the four field trials in the 2015-T, 2015-H, 2016-T, and 2016-H plantings. “KURT” and “SKEW” functions in Microsoft Office Excel were used to calculate the skewness and kurtosis (Liu K. et al., 2018). The differences of parental phenotypes were detected by the Student's *T*-test. The StatgenGxE package of R was used to test the significance of the genotypic and environment effects. The model of variance analysis is as follows: trait = trial + genotype + genotype: trial, in which trait is the phenotypic value, trial the environmental effect, genotype the genetic effect, genotype: trial the interaction effect between genotype and trial. Broad-sense heritability (*H*^2^) was calculated using the lme4 package of R based on the following formula:


$$\begin{array}{l} H^{2}=\frac{\sigma_{g}^{2\;}}{\sigma_{g}^{2\;}+\frac{\sigma_{ge}^{2\;}}{e}+\frac{\sigma_{\varepsilon }^{2}}{re}} \end{array}$$

where $\sigma_{\text{g}}^{2}$ is a variance component of genotype, $\sigma_{\text{ge}}^{2}$ is a variance component of genotype × environment interaction, $\sigma_{\varepsilon }^{2}$ is a variance component of random error, e is number of environments, and r is number of replicates per environment (Liu X. et al., 2018).

### Lignin Staining and SEM

Phloroglucinol-HCl staining was carried out as described previously (Tang et al., 2014). The third internode below the primary ear at the tasseling stage was sectioned with a double-edged razor blade and stained with 1% phloroglucinol (w/v) in 12% HCl for 5 min and immediately observed with a microscope under white light. The cross section of 3705I and LH277 stems was observed by using scanning electron microscopy (Sindhu et al., 2007). Approximately, 0.5-cm^3^ tissues containing rind of third internodes below the primary ear were cut free-hand by the razor blade and fixed with 3.5% glutaraldehyde at 4°C for at least 24 h. Samples were rinsed four times (20 min each) in 0.1-M sodium phosphate (pH 7.2) and then post-fixed in 1% osmium tetroxide for 1 h. After several washes with 0.2-M sodium phosphate (pH 7.2), the samples were dehydrated in a graded ethanol series (30, 50, 70, 80, 90, and 100%) and the critical point dried. Finally, the samples were mounted on an SEM aluminum stub with double-sided tape and sputter-coated with gold. Images were captured by using a JSM-6610LV scanning electron microscope at 5–10 kV.

### DNA Extraction and SSR Analyses

Genomic DNA was extracted from fresh leaves of F_2_ and F_2:3_ families following the standard CTAB method (Murray and Thompson, 1980). Based on the maize genome database (http://www.maizegdb.org), 864 simple sequence repeat (SSR) markers that were distributed throughout the maize genome (Chromosome 1 to Chromosome 10) were selected to screen for polymorphisms between the two parents. These primers were synthesized at Sangon (Shanghai, China). Agarose gel (4%) electrophoresis with 0.5 mg/L EB and Ultraviolet gel imager were used to detect the polymorphism of the PCR products. PCR-based polymorphic SSR markers were used for detecting the genotype of F_2_ and F_2:3_ populations.

### Linkage Analysis and QTL Mapping

Based on the genotypic data of F_2_ and F_2:3_, the software JoinMap 4.0 (Ooijen and Van, 2006) was used to construct a linkage map and calculate map distances in centiMorgen (cM) by using the Kosambi mapping function. QTL analysis was conducted with QTL IciMapping 4.0 (Li et al., 2008) based on the inclusive composite interval mapping method (ICIM), with a walk speed of 1 cM. For each of the datasets (2015-T, 2015-H, 2016-T, 2016-H), a putative QTL with a significant threshold was estimated by permutation tests with 1,000 times at *p* < 0.05, with a logarithm of odds (LOD) score > 2.5.

### Fine Mapping of QTL

For fine mapping *qRPR1-3* and *qRPR3-1*, two major QTLs on Chromosomes 1 and 3, respectively, we developed different types of markers Insertions/Deletions (InDels), Cleaved Amplified Polymorphic Sequence (CAPS) based on various sequence variations, which included InDels and Single Nucleotide Polymorphisms (SNPs) between 3705I and LH277 parents (Supplementary Tables 3, 5). Based on the initial QTL analysis, a progeny-based sequential fine-mapping strategy was adopted to dissect the QTL region (Ma et al., 2017). The progeny from BC_4_F_1_, BC_6_F_1_, and BC_8_F_1_, which were created by backcrossing to the low RPR parent 3705I, was screened for fine mapping *qRPR1-3* and *qRPR3-1*. In each recombinant-derived progeny population (BC_5_F_1_, BC_7_F_1_, and BC_9_F_1_ populations), homozygotes harboring the 3705I allele and heterozygotes harboring the LH277 allele were identified by using markers (Supplementary Tables 3, 5) within the region of *qRPR1-3* and *qRPR3-1*, the phenotypic difference of which was detected. Student's *T*-test (*p* < 0.05) was adopted to estimate the significance of RPR difference. The BC_6_F_1_ and BC_8_F_1_ populations were planted in Hainan. Because of the bad weather, the BC_6_F_1_ and BC_8_F_1_ populations were used exclusively for screening more recombinants.

### RNA Extraction and qRT-PCR

Stem rind tissues in V6, V8, V10, V12, and VT stages were collected from the third internodes below the primary ear of NIL-3705I and NIL-LH277. Total RNA was extracted from rind tissues by using a Trizol reagent (Thermo Fisher Scientific, USA). *Evo M-MLVPlus* 1st Strand cDNA Synthesis Kit (Accurate Biotechnology, Hunan, China) was used to synthesize cDNA from the total RNA. qRT-PCR with gene-specific primers was performed on an Applied Biosystems PCR instrument VIIA7 by using the UltraSYBR Mixture (Cwbio, China) according to the manufacturers' protocols (Supplementary Table 5). The expression levels of candidate genes were normalized to those of the *CULLIN2* control (Manoli et al., 2012).

## Results

### Selection of Maize Inbred Lines With Contrasting Stalk-Lodging Resistance

**Figure 1:**
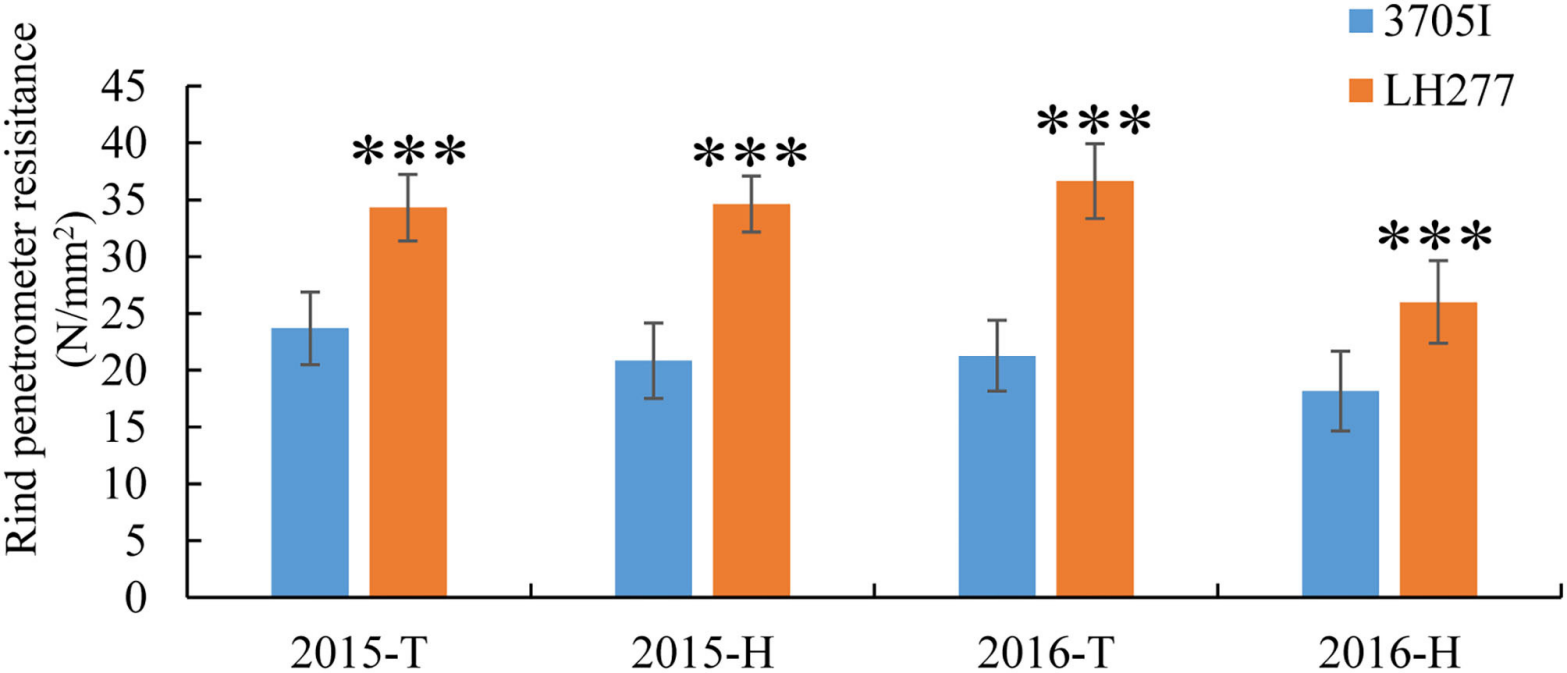
Rind penetrometer resistance of 3705I and LH277 in 2015 and 2016. ***Significant at *p* < 0.001, T, Tai'an; H, Hainan.

**Figure 2:**
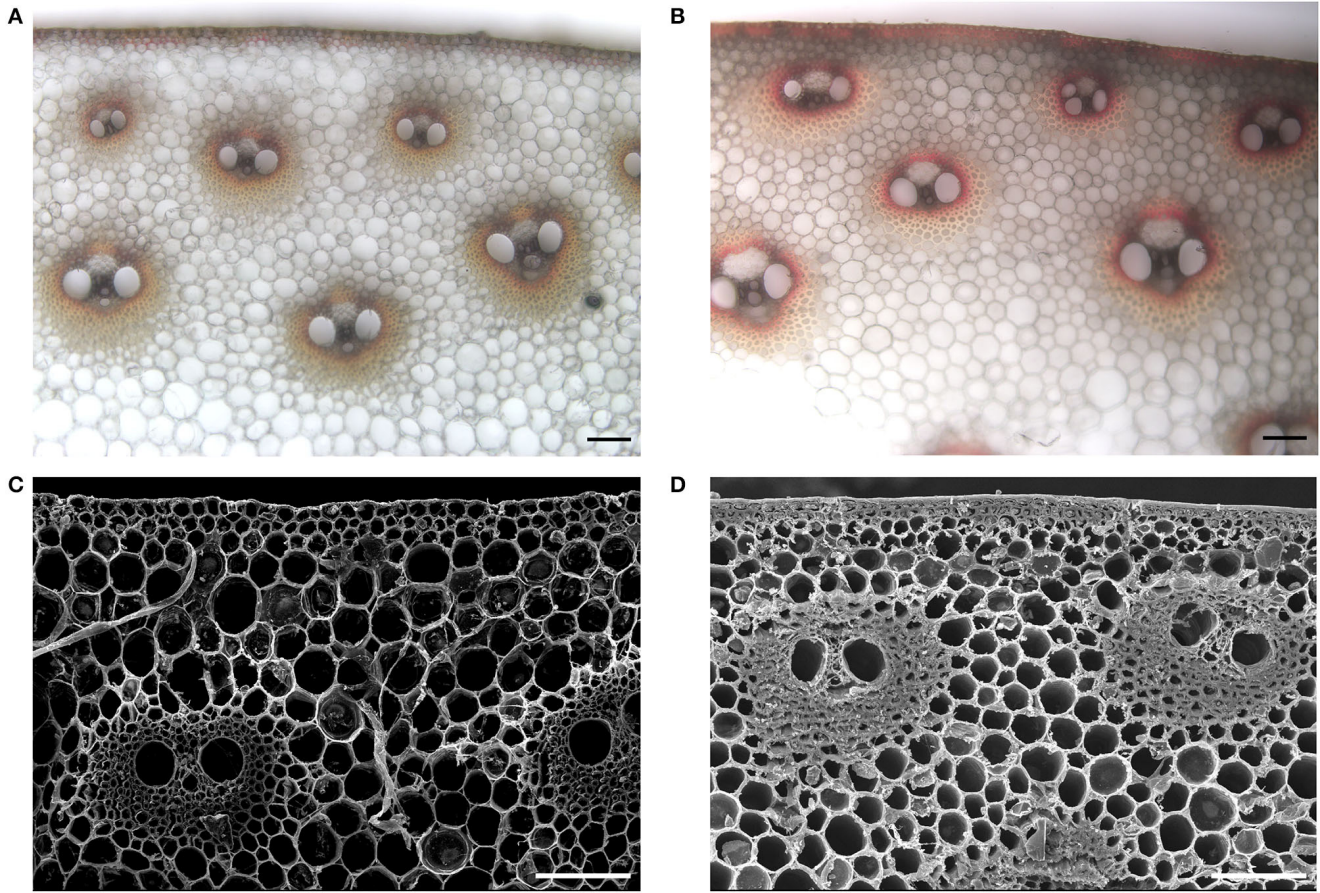
Morphological characteristic of cross-sections of the third internodes below the primary ear in 3705I and LH277. **(A,B)** Are phloroglucinol staining of lignin of 3705I stems and LH277 stems, respectively. A scale bar of **(A,B)**, 100 μm. **(C,D)** Are SEM images of cross-sections of 3705I stems and LH277 stems, respectively. A scale bar of **(C,D)**, 100 μm.

A small set of maize inbred lines was initially selected according to their performance of lodging resistance in the field. To further evaluate the capability of their stalk-lodging resistance quantitatively, rind penetrometer resistance (RPR) of them was measured at the tasseling stage in four growing seasons (Table 1). Among them, the line LH277 showed consistently high RPR, whereas the RPR of 3705I was consistently and significantly low (*p* < 0.001), suggestive of their opposite stalk-lodging resistance (Figure 1). Then, we stained the lignin of the third internodes below the primary ears of both lines and observed that the staining color was deeper in LH277 (Figure 2B) than in 3705I (Figure 2A) in the epidermis and around the vascular bundle, indicative of more lignin deposition in cell walls of LH277 (Figures 2A,B). To gain a detailed view of cell walls in stalk tissues of both lines, scanning electron microscopy (SEM) was used to observe the cross sections of their third internodes. The cell walls of epidermis and hypodermis in 3705I (Figure 2C) were much thinner than those in LH277 (Figure 2D), which indicated that there were more developed secondary cell walls in LH277. Similarly, the cell walls in the vascular bundles of LH277 (Figure 2D) were thicker than those of 3705I (Figure 2C). These results together suggested that the difference of cell wall thickness and the degree of lignin deposition between 3705I and LH277 may be mainly responsible for the difference of their RPR. Both of them were thus selected as parental lines for genetic population construction and QTL mapping of RPR.

**Table 1:** Phenotypic performance and broad-sense heritability for RPR in F_2_ and F_2:3_ populations.

**Populations**	**Environment**	**3705I**	**LH277**	**Populations**				
		**Mean ±SD**	**Mean ±SD**	**Mean ±SD**	**Range**	**Skewness**	**Kurtosis**	**95% Confidence** **interval (N/mm^**2**^)**	**H^**2**^ (%)**
		**(N/mm^**2**^)**	**(N/mm^**2**^)**	**(N/mm^**2**^)**	**(N/mm^**2**^)**				
F2	2015T	23.67 ± 3.19	34.30 ± 2.92***	30.36 ± 6.3	14.04–49.15	0.154698	−0.09134	21.32–40.11	72
F2	2015H	20.83 ± 3.31	34.62 ± 2.46***	36.90 ± 6.77	19.54–54.90	0.015809	−0.16317		
F2:3	2016T	21.27 ± 3.12	36.63 ± 3.29***	32.75 ± 5.10	19.09–48.00	0.367778	−0.07137		
F2:3	2016H	18.16 ± 3.50	26.00 ± 3.64***	24.86 ± 4.55	12.53–44.03	0.399844	1.023398		

*T, Tai'an; H, Hainan.*

***Significant at P <0.001.

### Phenotypic Analysis

**Figure 3:**
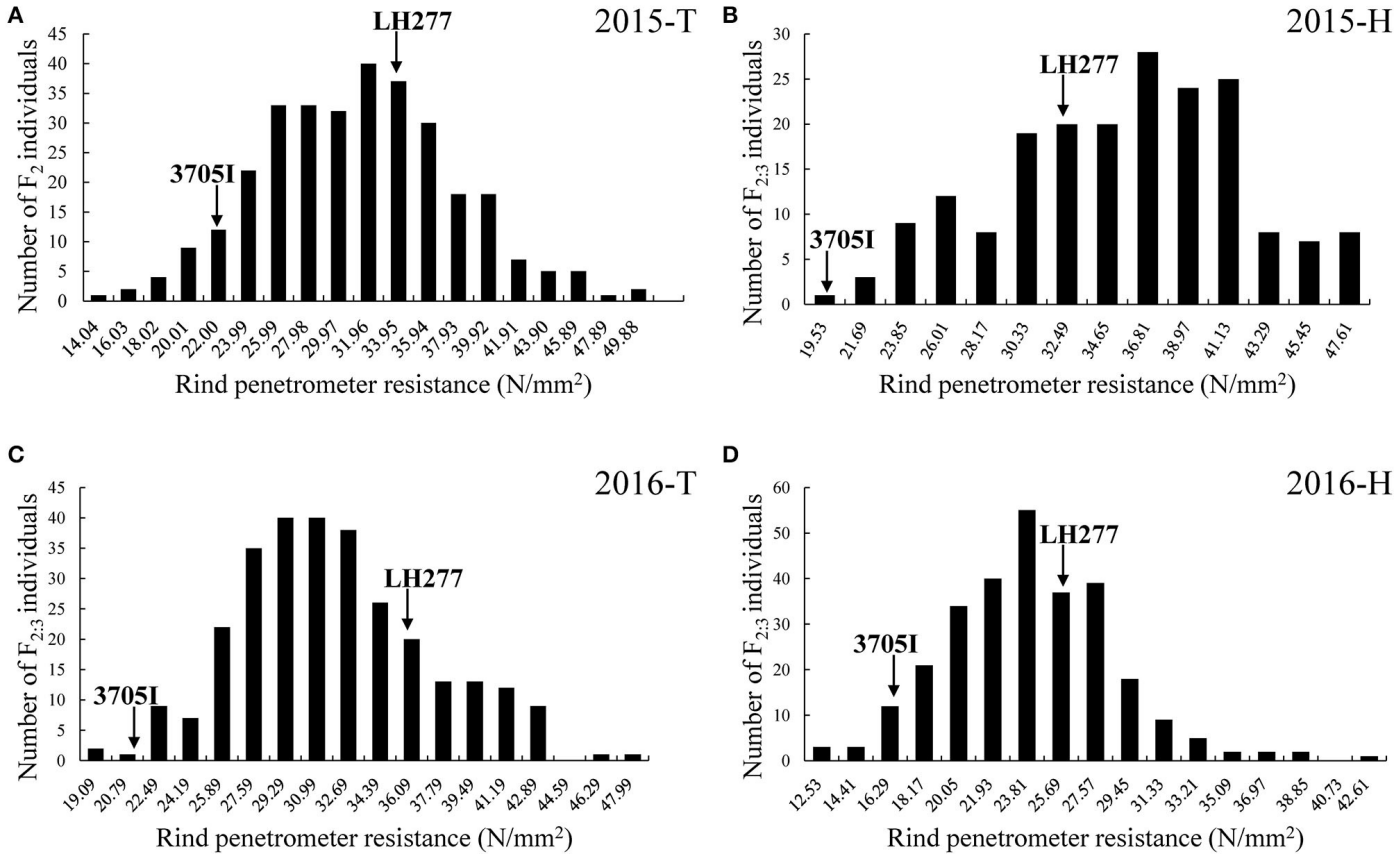
Frequency distribution of rind penetrometer resistance in the F_2_ and F_2:3_ populations, respectively. **(A)** RPR of F_2_ in 2015-T. **(B)** RPR of F_2_ in 2015-H. **(C)** RPR of F_2:3_ in 2016-T. **(D)** RPR of F_2:3_ in 2016-H. The x-axis indicates the rind penetrometer resistance, and the y-axis indicates the number of individuals in each group. In each trial, the two arrows represent the rind penetrometer resistance of both parental lines. T, Tai'an; H, Hainan.

Four field trials were conducted in four growing seasons. The mean RPR values of F_2_ families (30.36 ± 6.37 N/mm^2^) in 2015-T and F_2:3_ families (32.75 ± 5.10 N/mm^2^, 24.86 ± 4.55 N/mm^2^) in 2016-T and 2016-H were lower than the mean RPR value of both parental lines. And the mean RPR value of F_2:3_ families (36.90 ± 6.77 N/mm^2^) in 2015-H was higher than the mean RPR value of parental lines (Table 1). Wide phenotypic variation of RPR was detected, and the frequency distribution of RPR in F_2_ and F_2:3_ families exhibited a continuous variation, ranging from 12.53 to 54.90 N/mm^2^, with average values of 30.36, 36.90, 32.75, and 24.86 N/mm^2^, respectively (Figure 3). The skewness and kurtosis for RPR of F_2_ and F_2:3_ families indicated a normal distribution of RPR, suggesting that RPR is a typical quantitative trait controlled by polygenic loci. The variance was mainly derived from the different genotypes among F_2_ and F_2:3_ populations. At the same time, part of the variance derived from the interaction between genotypes and environment (Supplementary Table 1). The broad-sense heritability (*H*^2^) of RPR was estimated to be 72% (Table 1) across the four field trials, which means that genetics largely account for the lodging resistance of maize.

### QTL Analysis

All of 864 high-quality simple sequence repeat (SSR) markers across the maize whole genome were screened, of which 279 SSR markers (32.3% of the total) showed clear polymorphism between 3705I and LH277. After quality control, 267 SSR markers were used to genotype the 214 F_2_ individuals, and a linkage map was subsequently constructed (Supplementary Table 2). Ten linkage groups formed the linkage map, the total genetic length of which spanned 1,312.00 cM, with an average interval of 5.11 cM between adjacent markers.

In total, 14 QTLs for RPR were detected based on the F_2_ and F_2:3_ families across four growing seasons and distributed in eight chromosomes, except Chromosomes 2 and 6 (Table 2; Supplementary Figure 2). The additive and dominance effects were detected at all of the mapped RPR QTLs, with additive effects ranging from 0.05 to 2.67 and dominance effects ranging from 0.01 to 2.28. Each QTL could explain the phenotypic variation of RPR from 4.14% (*qRPR4*) to 15.89% (*qRPR1-3*). Of the RPR-increasing alleles at the 14 QTL loci, seven were derived from the high-RPR line LH277, and the other seven were derived from low-RPR line 3705I.

**Table 2:** QTL for rind penetrometer resistance in F_2_ and F_2:3_ populations.

**QTL^**a**^**	**Field trial**	**Population**	**Chr**	**Bin^**b**^**	**Marker interval**	**LOD^**c**^**	**PVE^**d**^**	**Add^**e**^**	**Dom^**f**^**
*qRPR1-1*	2015-T	F_2_	1	1.02-1.02	bnlg1953-umc1166	2.51	5.56	−1.96	0.25
*qRPR1-2*	2015-H	F_2:3_	1	1.05-1.05	umc1395-umc1297	5.51	9.57	−2.94	0.01
*qRPR1-3*	2016-T	F_2:3_	1	1.09-1.08	umc1306-mmc0041	7.30	14.05	−2.75	1.08
	2016-H	F_2:3_				6.88	15.89	−2.79	−1.49
*qRPR3-1*	2015-T	F_2_	3	3.03-3.04	bnlg1447-Phi036	5.99	12.25	−2.83	0.76
	2016-T	F_2:3_				6.29	10.27	−2.42	0.81
*qRPR3-2*	2015-H	F_2:3_	3	3.04-3.05	umc1717a-umc1907	4.33	7.08	−2.07	1.57
*qRPR3-3*	2016-T	F_2:3_	3	3.06-3.06	umc1951-bnlg1160	2.62	4.44	0.05	−2.31
*qRPR3-4*	2016-H	F_2:3_	3	3.05-3.05	umc1907-mmc0022	4.62	8.01	−1.82	0.73
*qRPR4*	2015-H	F_2:3_	4	4.05-4.04	umc2061-umc1652	2.68	4.14	−0.95	2.28
*qRPR5*	2015-H	F_2:3_	5	5.03-5.04	umc2161-umc1591	4.52	7.70	2.67	0.03
*qRPR7-1*	2015-T	F_2_	7	7.03-7.04	umc1936-umc1710	3.46	6.99	2.21	0.22
*qRPR7-2*	2016-T	F_2:3_	7	7.04-7.03	umc1710-bnlg339	3.14	5.86	1.76	−1.15
*qRPR8*	2016-T	F_2:3_	8	8.03-8.02	umc1157-umc1304	3.57	5.65	1.60	0.71
*qRPR9*	2015-H	F_2:3_	9	9.04-9.04	bnlg1209-umc2121	4.02	6.80	2.55	−0.10
*qRPR10*	2016-H	F_2:3_	10	10.05-10.04	umc2043-umc1930	4.41	8.37	1.86	−1.65

*^a^Name for QTL associated with an RPR trait.*

*^b^Bins are estimated based on the proximity of the QTL to the left flanking marker and the bin location of markers in the MaizeGDB.*

*^c^Logarithm of the odds.*

*^d^Phenotypic variation explained by the QTL.*

*^e^Additive effect.*

*^f^Dominance effect*.

Twelve RPR-associated QTLs were detected in only one field trial, and each of them only accounted for <10% phenotypic variation, indicating that these QTLs with small effect were highly sensitive to environment (Table 2). Two large-effect QTLs, *qRPR1-3* and *qRPR3-1*, were detected across two field trials. *qRPR1-3*, flanked by the umc1306 and mmc0041 markers on Chromosome 1, accounted for 14.05% (LOD = 7.30) and 15.89% (LOD = 6.88) of the total phenotypic variation of RPR in 2016-T and 2016-H, respectively (Table 2; Supplementary Figure 2). *qRPR3-1*, flanked by the bnlg1447 and Phi036 on Chromosome 3, accounted for 12.25% (LOD = 5.99) and 10.27% (LOD = 6.29) of the RPR variation in 2015-T and 2016-T. Both of them were thus chosen for fine mapping.

### Fine Mapping of *qRPR1-3*

**Figure 4:**
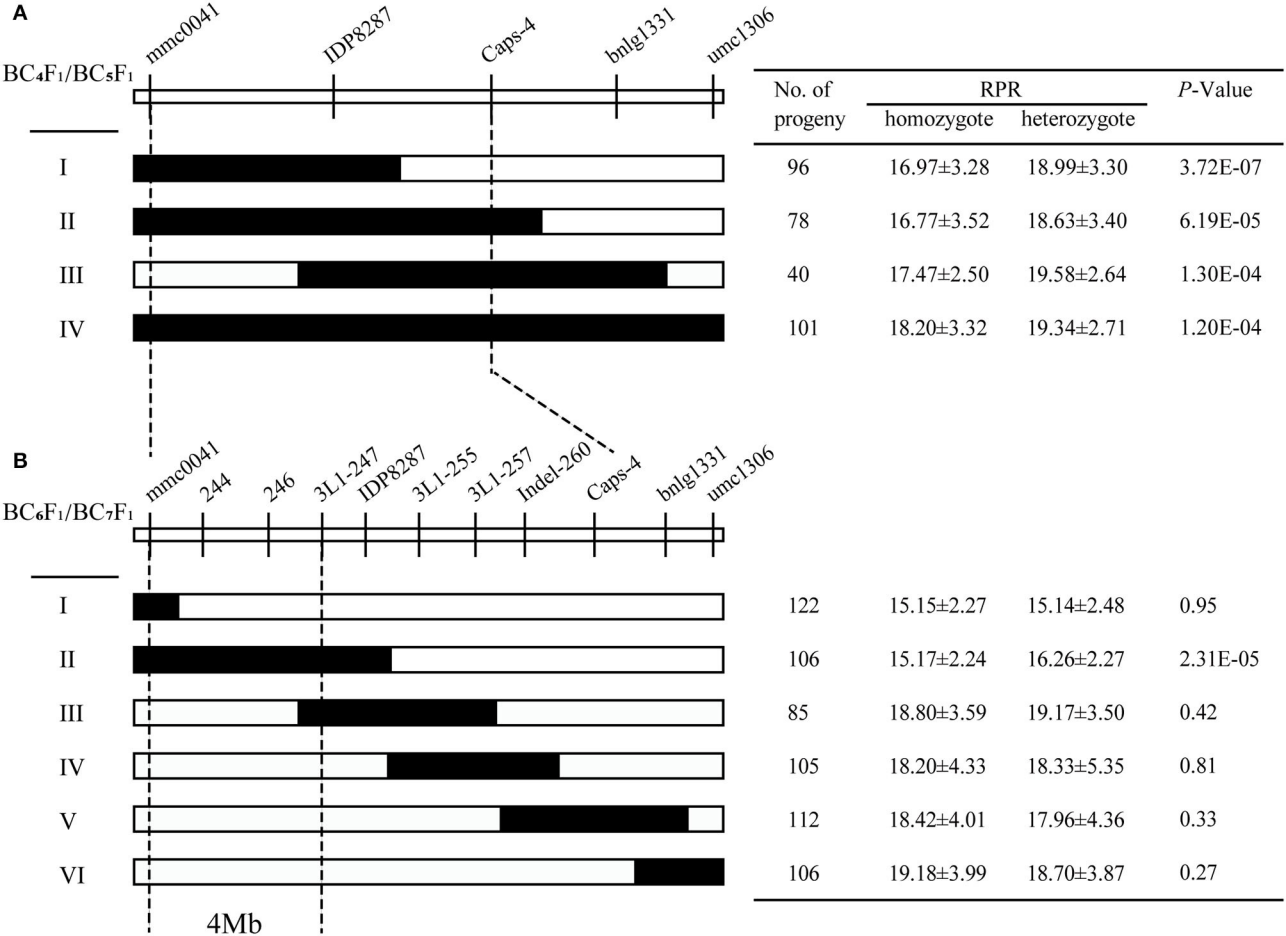
Fine mapping of *qRPR1-3*. Seven BC_4_F_1_ **(A)** and 19 BC_6_F_1_ **(B)** recombinants of *qRPR1-3* was classified into four and six types, respectively. In each recombinant type, the chromosomal composition of *qRPR1-3* is depicted as black or white, corresponding to heterozygous 3705I/LH277 and homozygous 3705I/3705I, respectively. The black-dotted lines indicate the narrowed regions of *qRPR1-3*. A significant difference (*p* < 0.05) between heterozygote 3705I/LH277 and homozygote 3705I/3705I indicated the presence of *qRPR1-3* in the donor region. The location of *qRPR1-3* was refined from a 26.8 Mb region to a 4 Mb region flanked by the markers mmc0041 and 3L1-247.

According to the initial QTL analysis, the confidence region covering the QTL *qRPR1-3* was corresponding with a physical distance of 26.8 Mb in the B73 reference genome (B73_RefGen_v4). For fine mapping of QTL *qRPR1-3*, 8 molecular markers were developed based on the B73 reference sequence, including one Cleaved Amplified Polymorphic Sequence (CAPS), and 7 Insertion/Deletion (InDel) markers (Supplementary Table 3). Two markers (mmc0041 and umc1306) flanking the *qRPR1-3* region and three markers (IDP8287, Caps-4, and bnlg1331) within it were used to screen the BC_4_F_1_ population, and seven recombinants were identified with crossover breakpoints in the *qRPR1-3* region. All the BC_5_F_1_ progeny of them (652 individuals) was used for RPR measurement. Progeny from Types I to IV exhibited a significant difference of RPR (*p* < 0.001) between homozygotes harboring the 3705I allele (16.77–18.20 N/mm^2^) and heterozygotes harboring the LH277 allele (18.63–19.58 N/mm^2^; Figure 4A), indicating the presence of *qRPR1-3* in the LH277 donor region. On the basis of this result, we could narrow the interval of *qRPR1-3* down to a 19 Mb window, which is flanked by markers mmc0041 and Caps-4 (Figure 4A).

In the next fine mapping of *qRPR1-3*, 11 markers (Supplementary Table 3) were used to screen the BC_6_F_1_ population and identify 19 recombinants in the *qRPR1-3* region. These 19 recombinants were classified into six types (I–VI; Figure 4B), and all BC_7_F_1_ progeny of them (1,990 individuals) was used for RPR measurement. Progeny from Type II, which carried a recombination event between mmc0041 and 3L1-255, showed significant difference (*p* < 0.001) of RPR between homozygotes (15.17 ± 2.24 N/mm^2^) and heterozygotes (16.26 ± 2.27 N/mm^2^; Figure 4B), indicating the presence of *qRPR1-3* in the LH277 donor region. Whereas, there was no significant difference (*P* > 0.05) of RPR between homozygotes (15.15–19.18 N/mm^2^) and heterozygotes (15.14–19.17 N/mm^2^), which were derived from the progeny of Types I, III, IV, V, and VI, indicating the absence of *qRPR1-3* in the LH277 donor region. Therefore, QTL *qRPR1-3* could be narrowed down to a 4Mb window between markers mmc0041 and 3L1-247 (Figure 4B).

### Fine Mapping of *qRPR3-1*

**Figure 5:**
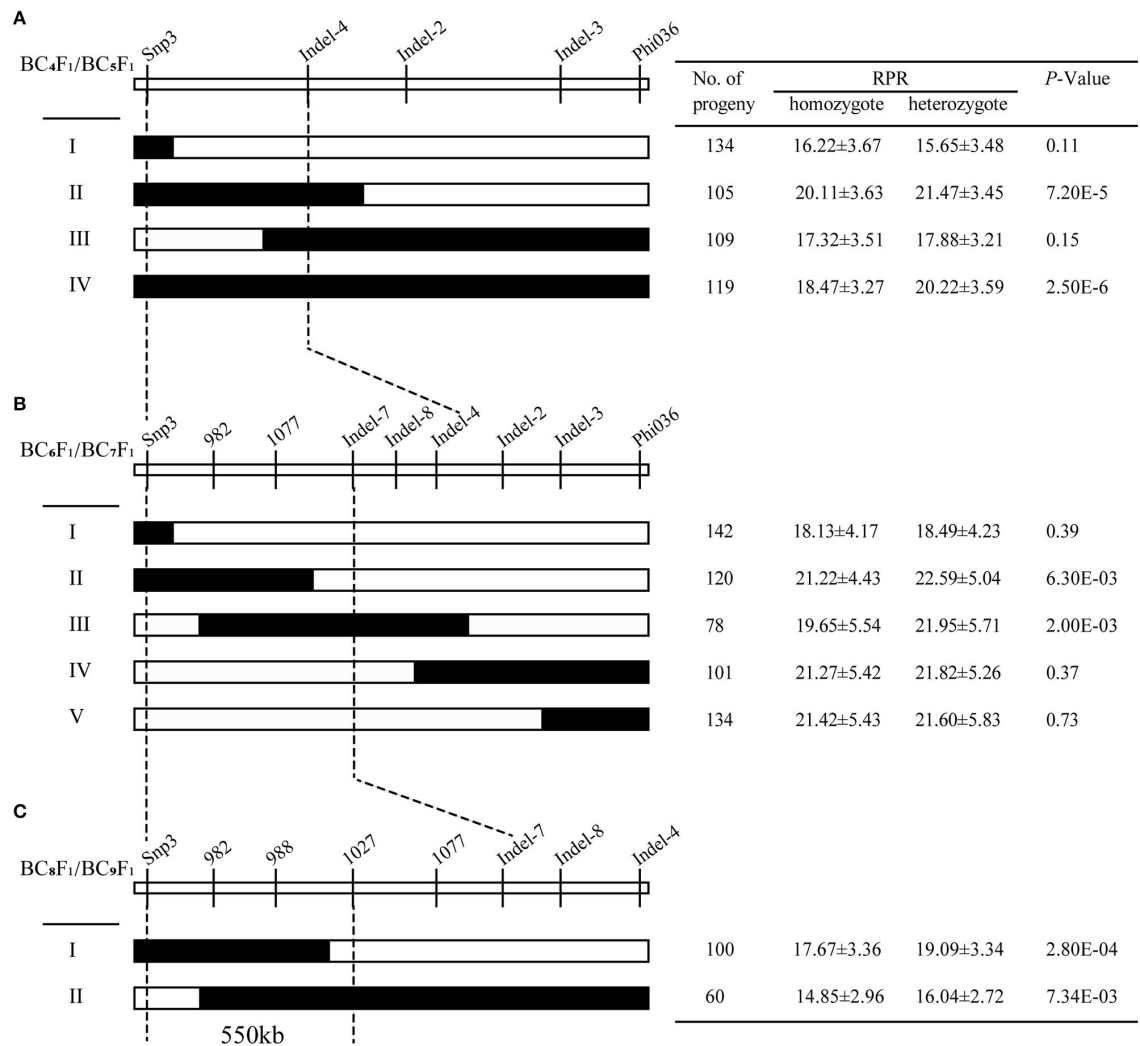
Fine mapping of *qRPR3-1*. Eight BC_4_F_1_ **(A)**, 14BC_6_F_1_ **(B)**, and six BC_8_F_1_ **(C)** recombinants of *qRPR3-1* were classified into four, five, and two types, respectively. In each recombinant type, the chromosomal composition of *qRPR3-1* is depicted as black or white, corresponding to heterozygous 3705I/LH277 and homozygous 3705I/3705I, respectively. The black-dotted lines indicate the narrowed regions of *qRPR3-1*. A significant difference (*p* < 0.05) between heterozygote 3705I/LH277 and homozygote 3705I/3705I indicated the presence of *qRPR3-1* in the donor region. The location of *qRPR3-1* was refined from a 4.5 Mb region to a 550 kb region flanked by the markers Snp3 and 1027.

For further resolving the position of *qRPR3-1*, 11 molecular markers that showed polymorphisms between two parents were developed in the 4.5 Mb QTL interval (Supplementary Table 5). Eight recombinants were screened from the BC_4_F_1_ population and classified into four types (I–IV; Figure 5A) by using markers Snp3 and Phi036 flanking and three markers (Indel-4, Indel-2, and Indel-3; Supplementary Table 5) within the interval of the *qRPR3-1* region. BC_5_F_1_ progeny (908 individuals) of recombinants was used for RPR measurement. Progeny from Types II and IV exhibited significant difference (*p* < 0.001) between homozygotes harboring the 3705I allele (20.11 ± 3.63 and 18.47 ± 3.27 N/mm^2^) and heterozygotes harboring the LH277 allele (21.47 ± 3.45 and 20.22 ± 3.59 N/mm^2^), indicating the presence of *qRPR3-1* in the LH277 donor region. Progeny from Types I and IV showed no significant difference (*p* > 0.05) of RPR between homozygotes (16.22 ± 3.67 and 17.32 ± 3.51 N/mm^2^) and heterozygotes (15.65 ± 3.48 and 17.88 ± 3.21 N/mm^2^), indicating the absence of *qRPR1-3* in the LH277 donor region (Figure 5A). Based on this result, *qRPR3-1* could be narrowed down to an interval of 1.8 Mb flanked by markers Snp3 and Indel-4.

In the next step of fine mapping, nine markers (Supplementary Table 5) were used to screen the BC_6_F_1_ population. Fourteen recombinants were identified in the *qRPR3-1* region and classified into five types (I–V; Figure 5B). BC_7_F_1_ progeny (1,566 individuals) of recombinants was used for RPR measurement. The RPR values of progeny derived from heterozygotes (21.95–22.59 N/mm^2^) were significantly higher (*p* < 0.01) than the RPR values of homozygotes (19.65–21.22 N/mm^2^), which were derived from progeny of Types II and III, indicating the presence of *qRPR3-1* in the LH277 donor region. Whereas, the homozygote (18.13–21.42 N/mm^2^) and heterozygote (18.49–21.82 N/mm^2^) of progeny from Types I, IV, and V showed similar RPR values (*p* > 0.05), indicating the absence of *qRPR3-1* in the LH277 donor region (Figure 5B). According to the above results, the target region of QTL *qRPR3-1* could be narrowed down to a 1 Mb window between markers Snp3 and Indel-7.

To fine map *qRPR3-1* further, six recombinants were identified from the BC_8_F_1_ population and were classified into two types (I–II; Figure 5C) by using eight markers within the interval of the *qRPR3-1* region. BC_9_F_1_ progeny (481 individuals) of the recombinants was used for RPR measurement. Progeny from Types I and II exhibited a significant difference of RPR (*p* < 0.01) between homozygotes (14.85–17.67 N/mm^2^) and heterozygotes (16.04–19.09 N/mm^2^) (Figure 5C), indicating the presence of *qRPR3-1* in the LH277 donor region. Therefore, QTL *qRPR3-1* could be narrowed down to a 550 kb window between markers Snp3 and 1027 (Figure 5C).

### Candidate Genes in the Target QTL Region of *qRPR3-1*

**Figure 6:**
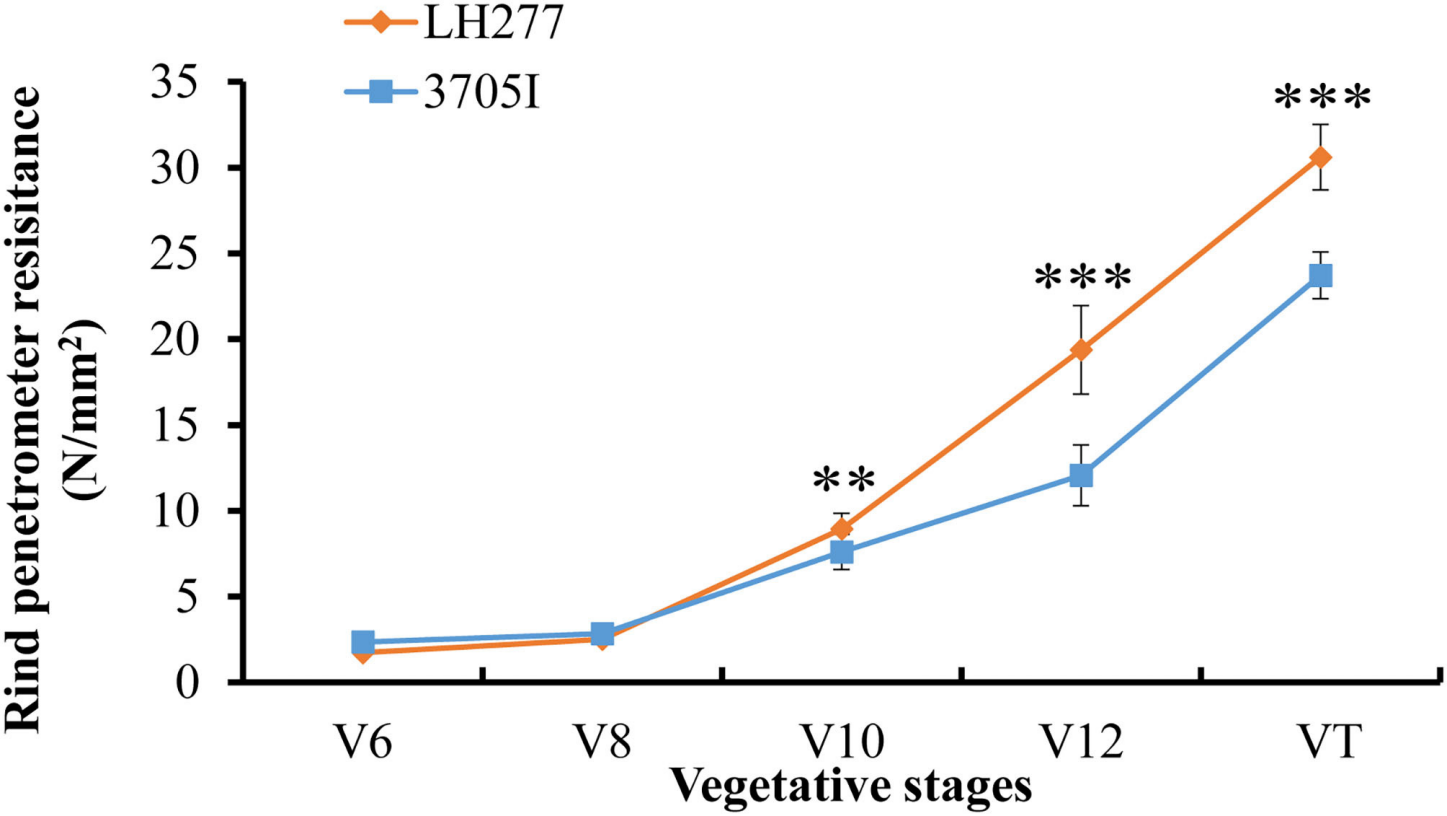
Rind penetrometer resistance of 3705I and LH277 in V6, V8, V10, V12, and VT stages. V6, V8, V10, and V12 represent the last leaf stages, which were defined according to the uppermost leaf whose collar is visible, and VT (tasseling) was the last vegetative stage. **Significant at *p* < 0.01, ***Significant at *p* < 0.001.

Based on the gene annotation of B73 reference sequence Version 4, there are 76 genes located in the 4 Mb target region of QTL *qRPR1-3* (Supplementary Table 4) and 12 genes located in the 550 kb target region of QTL *qRPR3-1* (Table 3). The candidate genes within *qRPR3-1* were further investigated because of their relatively small number. Given that the RPR was closely related with stalk development involving cell wall synthesis, the RPR of the third internodes of both parental lines in five vegetative stages was measured (Figure 6). In V6 and V8 stages, 3705I and LH277 showed similar RPR values. In V10, V12, and VT stages, however, the RPR values of LH277 were significantly higher than that of 3705I (*p* < 0.01, *p* < 0.001, and *p* < 0.001). These results suggested the RPR difference between 3705I and LH277 occurred in the late vegetative stages and increased along with the growth of plants, which was most likely associated with secondary cell wall growth, including lignin deposition.

**Table 3:** Candidate genes in the 550 kb physical region of *qRPR3-1*.

**Gene id**	**Chromosome**	**Gene start (bp)**	**Gene end (bp)**	**Protein length (AA)**	**Gene annotation**
*Zm00001d039634*	3	9,745,656	9,748,061	382	Dwarf plant1
*Zm00001d039635*	3	9,812,399	9,813,375	171	BES1/BZR1 homolog protein 4
*Zm00001d039636*	3	9,813,743	9,817,036	1097	Unknown function
*Zm00001d039637*	3	9,819,488	9,821,687	536	Myb family transcription factor EFM
*Zm00001d039638*	3	9,873,989	9,877,437	813	Unknown function
*Zm00001d039639*	3	9,879,223	9,883,575	464	Phototropic-responsive NPH3 family protein
*Zm00001d039640*	3	9,896,408	9,899,890	150	Double Clp-N motif-containing P-loop nucleoside triphosphate hydrolases superfamily protein
*Zm00001d039641*	3	9,974,966	9,976,264	432	Unknown function
*Zm00001d039642*	3	9,977,323	9,980,023	506	UDP-glycosyltransferase 73D1
*Zm00001d039643*	3	9,986,629	9,988,206	525	UDP-glycosyltransferase 73D1
*Zm00001d039644*	3	10,158,507	10,159,904	266	Cytokinin-O-glucosyltransferase 1
*Zm00001d039645*	3	10,161,178	10,161,525	115	Unknown function

**Figure 7:**
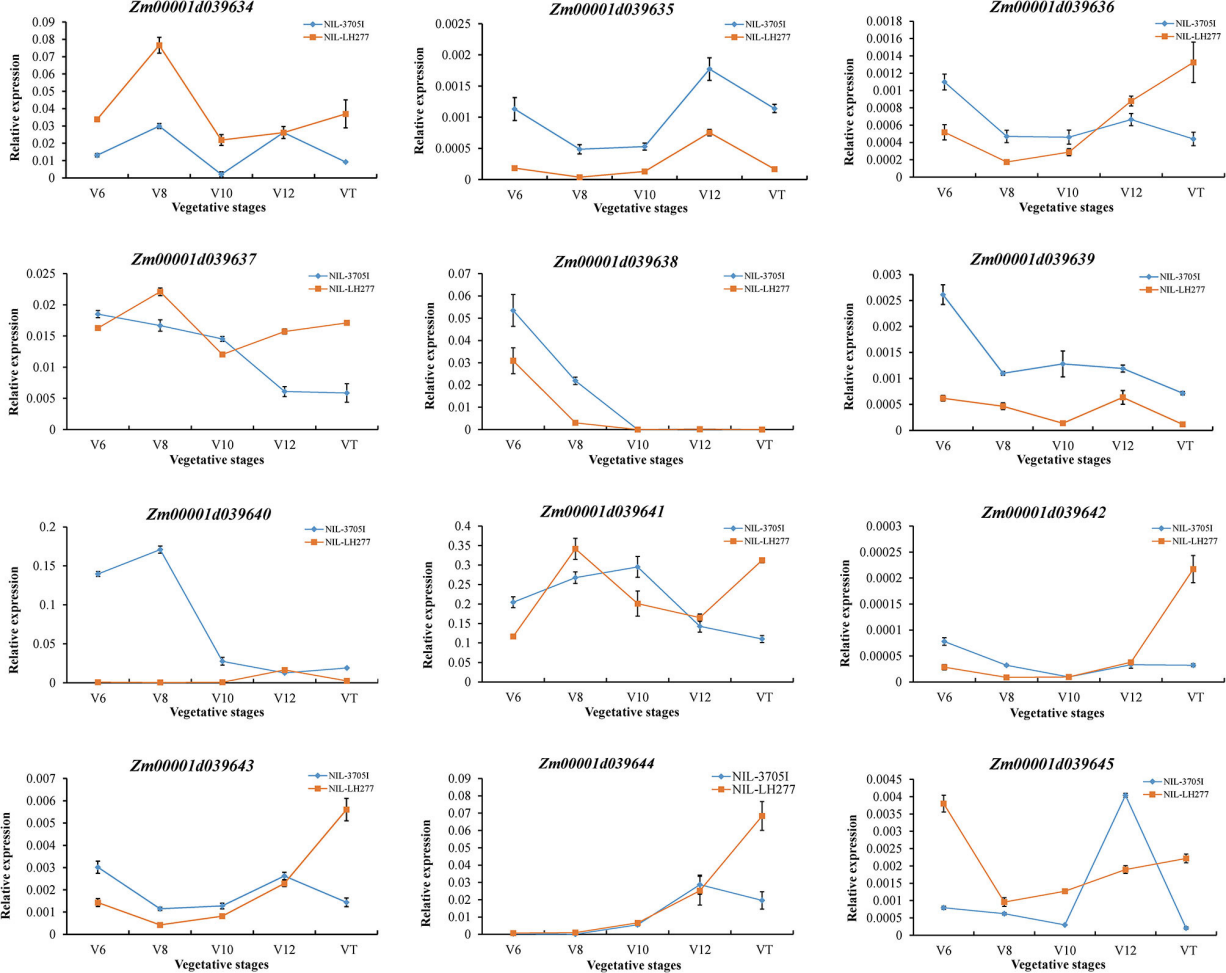
Relative expression levels of candidate genes in V6, V8, V10, V12, and VT stages. The expression levels of candidate genes were normalized to that of *CULLIN2*.

The expression of 12 candidate genes was then analyzed in five vegetative stages of NIL lines (Figure 7). *Zm00001d039638* and *Zm00001d039640* were barely expressed in V10, V12, and VT stages, inconsistent with the RPR difference. Compared to the moderately expressed reference gene *CULLIN2* (https://qteller.maizegdb.org/bar_chart_B73v4.php?name=Zm00001d024855), the expression level of genes, including *Zm00001d039635, Zm00001d039636, Zm00001d039639, Zm00001d039642, Zm00001d039643*, and *Zm00001d039645*, was extremely low, which makes these genes unlikely to be the causal genes for RPR difference. Therefore, one of *Zm00001d039634, Zm00001d039637, Zm00001d039641*, and *Zm00001d039644* might be responsible for the phenotypic difference, and all four of them need to be further investigated.

## Discussion

Accurate phenotypic measurement is crucial for QTL analysis (Cobb et al., 2013). The RPR of an internode at higher position below the primary ear of maize stalks is usually lower than those at lower positions (Gou et al., 2010). And RPR has been found to be negatively correlated with maize lodging (Twumasi-Afriyie and Hunter, 1982; Chesang-Chumo, 1993; Liu et al., 2020). In our study, the method of detecting RPR of third internodes below the primary ear was adopted to assess the maize lodging resistance in the tasseling stage (Supplementary Figure 1). The RPR of third internodes below the primary ear was significantly different between the inbred lines 3705I and LH277 (Figure 1). Although RPR as a complicated quantitative trait is controlled by genetics and environment heredity, genetic factors account for the phenotypic variation largely, and the broad-sense heritability of F_2_ and F_2:3_ populations of RPR was 72% (Table 1). The RPR performance of F_2_ and F_2:3_ populations (Figure 3) and the high broad-sense heritability of RPR verified that our adopted method was feasible for RPR QTL mapping. Compared to previous studies (Flint-Garcia et al., 2003a; Hu et al., 2012; Li et al., 2014), the reduced broad-sense heritability may be attributed to the difference of populations and growing environments.

By analyzing genotypes and phenotypes of F_2_ and F_2:3_ genetic populations across four field trials, 14 RPR QTLs were detected (Table 2; Supplementary Figure 2). Among them, the QTL *qRPR3-1* was also detected using NAM and IBM families, with which *qRPR1-1* was also detected at the same time (Peiffer et al., 2013). *qRPR10* was also detected by employing another population and accounted for 2.84% of the RPR variation (Hu et al., 2012). In addition to these three QTLs, other QTLs were detected for the first time in this study, including the largest-effect QTL *qRPR1-3*. The low repeatability of QTLs across different populations may be attributed to the complex genetic basis of maize RPR and their interaction with varied environments (Hu et al., 2012; Peiffer et al., 2013).

RPR as an indicator of maize stalk strength is highly correlated with the content of cell wall components, among which increased lignin, cellulose, and hemicellulose content can enhance maize stalk strength (Martin et al., 2004). In the internodes of the RIL population crossed with the inbred lines Ce03005 and B73, RPR was positively correlated with the content of some cell wall components, such as crude fiber, acid detergent lignin, acid detergent fiber, and cellulose in maize internodes (Hu et al., 2012). The suppression of *stiff1*, a gene located on Chromosome 6 underlying the major QTL for BS and RPR, led to the increase of cellulose and lignin content in maize stalk and, consequently, higher stalk strength (Zhang et al., 2020). The lignin content of third internodes in LH277 was higher than that in 3705I validated by lignin staining, while the cell walls of epidermis and vascular bundles in 3705I were much thinner than those in LH277 (Figure 2). Taken together, these results hinted that the RPR-associated QTLs were much likely to be related to cell wall components of maize stalk, and it was very likely that the causal genes are involved in the cell wall development or lignocellulose deposition.

By using a sequential fine-mapping strategy based on the progeny test, two major QTLs, *qRPR1-3* and *qRPR3-1*, were, respectively, narrowed down to 4 Mb and 550 kb physical intervals (Figures 4, 5), within which there are 76 and 12 genes (Supplementary Table 4; Table 3). There are too many candidate genes located within the target region of QTL *qRPR1-3* for screening, and we expect to do the corresponding work of candidate gene screening after further narrowing the interval of QTL *qRPR1-3*. The candidate genes within *qRPR3-1* were further investigated by using the qRT-PCR assay because of their relatively small number (Figure 7). By analyzing the expression pattern and the level of 12 candidate genes, *Zm00001d039634, Zm00001d039637, Zm00001d039641*, and *Zm00001d039644* are considered to be the most likely causal genes for RPR difference. The function of *Zm00001d039641* is unknown. *Zm00001d039634* encodes a gibberellin 3-oxidase to catalyze the final step of bioactive gibberellin synthesis, whereas gibberellin has been shown to regulate the development of stalks (Okuno et al., 2014; Peng et al., 2014). *Zm00001d039637* encodes a MYB transcription factor, and it has been reported that a subset of MYB transcription factors, such as *AtMYB46* and *AtMYB83*, orchestrates the biosynthesis of the plant secondary wall (McCarthy et al., 2009; Zhao and Dixon, 2011; Guo et al., 2017). *Zm00001d039644* encodes cytokinin-o-glucosyltransferase, rendering cytokinin inactivation temporarily, and cytokinin has been proved to be involved in regulation of flowering time (D'Aloia et al., 2011) and development of stalks (Nguyen et al., 2016). Thus, any of these three genes could not be excluded without further evidence. Moreover, all 12 candidate genes were retrieved from the B73 reference sequence Version 4. There may be extra and different candidate genes within the *qRPR3-1* interval of 3705I or the LH277 genome, given that more and more pangenes are identified (Hufford et al., 2021). Further fine mapping, genome segment sequencing, together with mutant analysis, are thus required for the identification and confirmation of the target gene. The functional characterization of the target gene would then unveil the role of the causal gene in regulating maize RPR.

In the maize breeding, stalk strength plays an important role in maintaining grain yield. In many maize synthetic populations, stalk strength has been successfully enhanced based on phenotypic selection for RPR (Masole, 1993; Zhang et al., 2020; Kumar et al., 2021). Marker-assisted selection (MAS) as an alternative way could improve the target agronomic traits in maize varieties, making breeding more efficient and rapid (Zhao et al., 2012; Liu et al., 2015; Kage et al., 2016). In this study, the major QTL *qRPR3-1* was narrowed down to the 550 kb window, containing 12 genes, and the use of four markers (Snp3, 982, 988, and 1027) allowed us to precisely introgress the *qRPR3-1* locus into a target maize inbred line for enhancing stalk strength. The small *qRPR3-1* location interval reduces the probability of a linkage drag of deleterious alleles. However, the genetic effects of some QTLs are more or less influenced by a genetic background, in particular mapping population (Liao et al., 2001; Li et al., 2017). Thus, the application of *qRPR3-1* on RPR selection should be further tested in more varieties.

## Data Availability Statement

The original contributions presented in the study are included in the article/Supplementary Material, further inquiries can be directed to the corresponding authors.

## Author Contributions

CC designed and supervised this project. XiH and SC conducted the experiments. XiH, SC, SW, TY, YW, PX, XX, QZ, and XuH performed the phenotypic data collection and field experiments. XiH, SC, and XuH carried out the genotypic data generation. XiH, SC, and TY contributed to the data analysis. XiH wrote the manuscript. GZ revised the manuscript. All authors contributed to the article and approved the submitted version.

## Funding

This research was supported by the Key Project of Shandong Natural Science Foundation, China to CC (ZR2020KC041), the Young Scientists Fund of the National Natural Science Foundation of China to GZ (31901556), and the National Key Research and Development Program of China to CC (2017YFD0101200 and 2016YFD0101003).

## Conflict of Interest

The authors declare that the research was conducted in the absence of any commercial or financial relationships that could be construed as a potential conflict of interest.

## Publisher's Note

All claims expressed in this article are solely those of the authors and do not necessarily represent those of their affiliated organizations, or those of the publisher, the editors and the reviewers. Any product that may be evaluated in this article, or claim that may be made by its manufacturer, is not guaranteed or endorsed by the publisher.
