# Supplementary material for: Characterization and Fine Mapping of qRPR1-3 and qRPR3-1, Two Major QTLs for Rind Penetrometer Resistance in Maize
Source: Front Plant Sci. 2022 Jul 19;13:944539. doi: 10.3389/fpls.2022.944539 (PMC9344970; doi:10.3389/fpls.2022.944539)
Supplement: Supplementary file 1 [file Data_Sheet_1.docx]

Supplemental Table S1 Analysis of variance for RPR in F_2_ and F_2:3_ populations

| Source | *df* | Sum of squares | Mean squares | *f* value | Pr > *f* | Variance |
| --- | --- | --- | --- | --- | --- | --- |
| Trial | 2 | 459.2 | 459.19 | 22.7790 | 3.007e-06 ^***^ | 0.0462 |
| Genotype | 265 | 12738.1 | 48.07 | 2.3845 | 1.703e-12 ^***^ | 0.3902 |
| Residuals | 530 | 5342.0 | 20.16 |  |  | 0.5637 |

*df*: degrees of freedom, *** significant at *P* < 0.001

Supplemental Table S2 Summary of the linkage map characteristics of the F_2_ population

| Molecular marker | Number of loci in each chromosome | | | | | | | | | |  |  |
| --- | --- | --- | --- | --- | --- | --- | --- | --- | --- | --- | --- | --- |
|  | 1 | 2 | 3 | 4 | 5 | 6 | 7 | 8 | 9 | 10 | Total loci | |
| SSR | 38 | 33 | 36 | 23 | 28 | 22 | 24 | 20 | 22 | 21 | 267 | |
| Length (cM) | 221.76 | 149.84 | 168.34 | 116.91 | 87.80 | 132.38 | 113.65 | 98.50 | 115.51 | 107.32 | 1312.00 | |
| Avg. interval (cM) | 5.99 | 4.68 | 4.81 | 5.31 | 3.25 | 6.30 | 4.94 | 5.18 | 5.50 | 5.37 | 5.11 | |
| Min. interval (cM) | 0.13 | 0 | 0 | 0 | 0 | 0 | 0.17 | 0.01 | 0 | 0.31 |  | |
| Max. interval (cM) | 16.75 | 21.28 | 17.47 | 25.32 | 14.67 | 16.11 | 22.79 | 14.05 | 16.71 | 11.73 |  | |

Supplemental Table S3 The primers of *qRPR1-3* fine mapping markers

| Marker | Type | Chromosome | Physical position (Mb) | Forward primer | Reverse primer |
| --- | --- | --- | --- | --- | --- |
| mmc0041 | SSR | 1 | 243,206,471-243,206,645 | AGGACTTAGAGAGGAAACGAA | TTTATCCTTACTTGCAGTTGC |
| 244 | InDel | 1 | 244,739,495-244,739,616 | GTCCACACGTTATTTGCGTG | ACATGAACAATATGACAAAGTG |
| 246 | InDel | 1 | 246,851,143-246,851,279 | AGCTCGAGCACGAGGCTAAA | CGAGCTAATTTCCAGCCTAT |
| 3L1-247 | InDel | 1 | 247,744,203-247,744,317 | AACATTGCCAAGGGAAAGCG | GGACATGGCTGCTTCCCTAG |
| IDP8287 | InDel | 1 | 249,400,315-249,400,772 | GAGGAAGACGTTCACTTCGG | TTCACCTTCGGCTTGTTACC |
| 3L1-255 | InDel | 1 | 255,038,734-255,039,198 | ATGGGGCCTCAAGACAATGG | AAAGAATGGAGGCCCTGTGG |
| 3L1-257 | InDel | 1 | 257,128,806-257,129,140 | GCACAAGAGTTGCTTCAGCC | TATCCTCGCATCGCAGGAAC |
| Indel-260 | InDel | 1 | 260,533,059-260,533,421 | GTGACGAGTACCCGGATTGG | GAAGGAGCACGCCATTGTTG |
| Caps-4 | CAPS | 1 | 262,998,362-262,998,982 | CGAAATGGTGAACTCTGATAC | GCCCCAAGAAAACCACTCAG |
| bnlg1331 | SSR | 1 | 263,894,488-263,894,714 | TGGTGATAACTGTCAAGCGC | TTGGGGCATTGGCCTATATA |
| umc1306 | SSR | 1 | 269,988,356-269,988,490 | CCAGGATGAATAAATCGTATTGCC | CGAAACAAAACACCCAGCAGTAGT |

Supplemental Table S4 Candidate genes in the 4Mb physical region of *qRPR1-3*

| Gene id | Chromosome | Gene start (bp) | Gene end (bp) | Protein length (AA) | Gene annotation |
| --- | --- | --- | --- | --- | --- |
| *Zm00001d032917* | 1 | 243,021,344 | 243,024,764 | 168 | Unknown function |
| *Zm00001d032918* | 1 | 243,021,464 | 243,022,587 | 311 | Unknown function |
| *Zm00001d032919* | 1 | 243,027,744 | 243,032,761 | 522 | Osmotin-like protein |
| *Zm00001d032921* | 1 | 243,105,233 | 243,132,987 | 629 | Unknown function |
| *Zm00001d032922* | 1 | 243,201,405 | 243,204,865 | 436 | Zinc finger protein ID1 |
| *Zm00001d032923* | 1 | 243,208,029 | 243,211,663 | 357 | Heat stress transcription factor A-6b |
| *Zm00001d032925* | 1 | 243,297,436 | 243,315,080 | 182 | 1, 2-dihydroxy-3-keto-5-methylthiopentene dioxygenase 4 |
| *Zm00001d032926* | 1 | 243,418,415 | 243,419,837 | 346 | Chlorophyllase2 |
| *Zm00001d032927* | 1 | 243,487,272 | 243,487,987 | 161 | Unknown function |
| *Zm00001d032929* | 1 | 243,530,719 | 243,532,173 | 206 | Unknown function |
| *Zm00001d032931* | 1 | 243,611,882 | 243,614,999 | 367 | Oxidoreductases |
| *Zm00001d032932* | 1 | 243,649,592 | 243,651,383 | 428 | IQ-domain 19 |
| *Zm00001d032933* | 1 | 243,735,038 | 243,744,089 | 2572 | Protein NETWORKED 1D |
| *Zm00001d032934* | 1 | 243,891,052 | 243,894,369 | 499 | Pentatricopeptide repeat-containing protein |
| *Zm00001d032935* | 1 | 243,995,654 | 244,000,344 | 591 | Type IV inositol polyphosphate 5-phosphatase 7 |
| *Zm00001d032937* | 1 | 244,096,829 | 244,098,160 | 282 | FCS-Like Zinc finger 8 |
| *Zm00001d032938* | 1 | 244,115,527 | 244,115,853 | 108 | Unknown function |
| *Zm00001d032939* | 1 | 244,122,165 | 244,125,272 | 190 | Unknown function |
| *Zm00001d032942* | 1 | 244,208,637 | 244,210,392 | 371 | TD and POZ domain-containing protein 1 |
| *Zm00001d032943* | 1 | 244,208,939 | 244,210,054 | 371 | Speckle-type POZ protein |
| *Zm00001d032944* | 1 | 244,267,423 | 244,291,732 | 369 | Short integuments 2 mitochondrial |
| *Zm00001d032945* | 1 | 244,341,397 | 244,343,019 | 493 | Unknown function |
| *Zm00001d032946* | 1 | 244,382,684 | 244,383,532 | 282 | Chitinase 1 |
| *Zm00001d032947* | 1 | 244,392,841 | 244,393,701 | 286 | Chitinase 1 |
| *Zm00001d032948* | 1 | 244,409,830 | 244,413,837 | 495 | 3-ketoacyl-CoA synthase |
| *Zm00001d032949* | 1 | 244,466,506 | 244,468,199 | 56 | Unknown function |
| *Zm00001d032950* | 1 | 244,489,885 | 244,492,952 | 380 | GDP-mannose 35-epimerase |
| *Zm00001d032954* | 1 | 244,535,139 | 244,536,005 | 39 | Unknown function |
| *Zm00001d032955* | 1 | 244,544,978 | 244,546,492 | 300 | Unknown function |
| *Zm00001d032956* | 1 | 244,604,888 | 244,627,143 | 826 | Acylamino-acid-releasing enzyme |
| *Zm00001d032957* | 1 | 244,726,721 | 244,727,637 | 223 | Thymidylate kinase |
| *Zm00001d032958* | 1 | 244,779,992 | 244,791,263 | 131 | Unknown function |
| *Zm00001d032960* | 1 | 244,814,311 | 244,818,194 | 157 | Unknown function |
| *Zm00001d032961* | 1 | 244,858,795 | 244,867,417 | 890 | ent-copalyl diphosphate synthase AN1 |
| *Zm00001d032962* | 1 | 244,881,980 | 244,894,327 | 617 | Myosin-binding protein 1 |
| *Zm00001d032964* | 1 | 244,930,865 | 244,933,652 | 356 | Lysine-ketoglutarate reductase/saccharopine dehydrogenase bifunctional enzyme |
| *Zm00001d032967* | 1 | 244,981,884 | 244,997,475 | 357 | Unknown function |
| *Zm00001d032968* | 1 | 244,998,348 | 245,014,854 | 341 | Tetrapyrrole (Corrin/Porphyrin) Methylases |
| *Zm00001d032969* | 1 | 245,012,635 | 245,014,564 | 261 | Probable glutathione S-transferase BZ2 |
| *Zm00001d032970* | 1 | 245,040,432 | 245,046,336 | 162 | Unknown function |
| *Zm00001d032971* | 1 | 245,052,325 | 245,054,453 | 406 | Protein DETOXIFICATION 16 |
| *Zm00001d032972* | 1 | 245,113,943 | 245,114,809 | 288 | Pathogenesis-related thaumatin superfamily protein |
| *Zm00001d032973* | 1 | 245,143,521 | 245,150,786 | 521 | Glycerol-3-phosphate acyltransferase 5 |
| *Zm00001d032974* | 1 | 245,150,815 | 245,171,898 | 372 | Probable prolyl 4-hydroxylase 12 |
| *Zm00001d032975* | 1 | 245,171,720 | 245,172,358 | 212 | Unknown function |
| *Zm00001d032976* | 1 | 245,195,219 | 245,195,980 | 253 | Unknown function |
| *Zm00001d032977* | 1 | 245,224,996 | 245,246,546 | 113 | Unknown function |
| *Zm00001d032978* | 1 | 245,437,954 | 245,453,257 | 155 | Unknown function |
| *Zm00001d032979* | 1 | 245,560,265 | 245,573,773 | 605 | Carbon catabolite repressor protein 4 homolog 1 |
| *Zm00001d032980* | 1 | 245,685,122 | 245,715,000 | 1240 | Phospholipid-transporting ATPase 3 |
| *Zm00001d032981* | 1 | 245,736,073 | 245,740,635 | 656 | IQ domain-containing protein IQM2 |
| *Zm00001d032982* | 1 | 245,762,608 | 245,763,489 | 171 | Glutathione S-transferase family protein |
| *Zm00001d032983* | 1 | 245,782,719 | 245,783,360 | 106 | Peptidase family M48 family protein |
| *Zm00001d032984* | 1 | 245,784,840 | 245,795,190 | 327 | Alanine aminotransferase 2 mitochondrial |
| *Zm00001d032985* | 1 | 245,797,940 | 245,803,282 | 1609 | ATP-dependent DNA helicase |
| *Zm00001d032986* | 1 | 245,816,492 | 245,820,070 | 465 | Unknown function |
| *Zm00001d032987* | 1 | 245,824,248 | 245,834,289 | 1829 | ATP-dependent DNA helicase |
| *Zm00001d032988* | 1 | 245,832,298 | 245,832,753 | 151 | Unknown function |
| *Zm00001d032989* | 1 | 245,837,747 | 245,855,929 | 387 | Serine/threonine-protein phosphatase |
| *Zm00001d032990* | 1 | 245,857,450 | 245,866,245 | 183 | SET domain protein123 |
| *Zm00001d032991* | 1 | 246,031,572 | 246,034,941 | 437 | F-box/kelch-repeat protein |
| *Zm00001d032992* | 1 | 246,072,019 | 246,074,488 | 324 | Pectinesterase 31 |
| *Zm00001d032993* | 1 | 246,131,231 | 246,140,049 | 198 | Unknown function |
| *Zm00001d032994* | 1 | 246,212,812 | 246,218,892 | 514 | Probable mannan synthase 7 |
| *Zm00001d032997* | 1 | 246,334,848 | 246,342,784 | 51 | PTI1-like tyrosine-protein kinase 3 |
| *Zm00001d032999* | 1 | 246,355,067 | 246,357,254 | 450 | DOF40 C2C2-DOF type transcription factor |
| *Zm00001d033002* | 1 | 246,561,191 | 246,566,041 | 525 | pyrophosphate--fructose 6-phosphate 1-phosphotransferase |
| *Zm00001d033003* | 1 | 246,635,889 | 246,636,588 | 116 | Unknown function |
| *Zm00001d033004* | 1 | 246,673,838 | 246,679,239 | 436 | Protein kinase 2B chloroplastic |
| *Zm00001d033005* | 1 | 246,790,551 | 246,792,861 | 363 | Homeobox-leucine zipper protein HAT7 |
| *Zm00001d033007* | 1 | 246,807,377 | 246,811,927 | 271 | Fringe-related protein |
| *Zm00001d033008* | 1 | 246,812,393 | 246,813,247 | 95 | 60S ribosomal protein L22-2 |
| *Zm00001d033009* | 1 | 246,830,535 | 246,831,469 | 76 | Unknown function |
| *Zm00001d033011* | 1 | 246,870,441 | 246,874,115 | 508 | Sucrose transporter |
| *Zm00001d033012* | 1 | 246,988,429 | 246,989,769 | 446 | F-box/kelch-repeat protein SKIP11 |
| *Zm00001d033013* | 1 | 247,039,907 | 247,040,320 | 137 | Unknown function |

Supplemental Table S5 The primers of *qRPR3-1* fine mapping markers

| Marker | Type | Chromosome | Physical position (Mb) | Forward primer | Reverse primer |
| --- | --- | --- | --- | --- | --- |
| Snp3 | CAPS | 3 | 9,729,491-9,730,490 | ATTGGCCAGACGGATCAAGG | CAGCTACTGGGGTAGGCATG |
| 982 | InDel | 3 | 9,827,010-9,827,162 | TCTCTCTATGTTTGGCTGAC | GACTCGTGTCAAGAGGAGAA |
| 988 | InDel | 3 | 9,882,415-9,882,549 | TTTACCCCCTGCACGAAAA | TTTTAGACGCTGGCTGGAA |
| 1027 | InDel | 3 | 10,270,577-10,270,723 | AATTGTCCACGTGCCTAGA | TGTAAAGAACGACTCGATGC |
| 1077 | InDel | 3 | 10,770,581-10,770,701 | AGATCGACGAGGAGGAGTGG | TGGTAAGCCCGCTACAAAGG |
| Indel-7 | InDel | 3 | 10,866,277-10,866,505 | GAACACTTGGATTCCCTTTG | TGCGGTTTATCCTAAGTCTT |
| Indel-8 | InDel | 3 | 11,524,777-11,524,972 | CTGAAACCAACTACTACTCTAT | CTTGTAATGTGACTCTTGGTG |
| Indel-4 | InDel | 3 | 11,806,596-11,807,414 | TTGTGAGAGTGAGAGGCATG | GCATCAGCTTCTCCTCCGAT |
| Indel-2 | InDel | 3 | 12,779,944-12,780,176 | TGGCCTACCACCAAGAACAC | TGCCCACCTACCTACTCCTG |
| Indel-3 | InDel | 3 | 13,213,332-13,213,604 | GGTGATGGATGGCACTTTAAC | CTCCTCCTGCTGCTAATCAC |
| Phi036 | SSR | 3 | 14,221,485-14,221,556 | CCGTGGAGAGACGTTTGACGT | TCCATCACCACTCAGAATGTCAGTGA |

Supplemental Table S6 Gene-specific primers for qRT-PCR

| Gene^a^ | Forward primer | Reverse primer |
| --- | --- | --- |
| *Zm00001d039634* | GCTTCTGTGATGTGATGG | CATGAGGAACAGCTCCAG |
| *Zm00001d039635* | AGGAGCAGCAGCATCAGG | ATGTAGGCGAAGGAAAGGG |
| *Zm00001d039636* | AGTCATCTCTTCTCAACGTGTC | GATCAGGTACATCCGTCCATTC |
| *Zm00001d039637* | GAAAGGGATGAGAATGTC | ACAAGTAAGGATTGCTAAT |
| *Zm00001d039638* | AATATGGCTATGAGGATTC | CTACACAGACTACAACAA |
| *Zm00001d039639* | GACACTCACGCAACAATTAGTT | CGCGAGGGTGTAGTAATAGTAG |
| *Zm00001d039640* | TGAGTAACACGCTCAATCTGAT | CCTGGTATACTCACAGACGTAG |
| *Zm00001d039641* | AAGAAGAGGAAGGTGACT | CTGCTGTCTCCATTGATA |
| *Zm00001d039642* | CATAAACAGCTTCCAAGAGCTG | CATACTGCCGAAGCTTACAAAG |
| *Zm00001d039643* | AAGGCAGCCATGGAGGAAG | CGTGGCTCTTCTTCATGTTTAG |
| *Zm00001d039644* | GAAGGTAACCATGATGGAAGGT | TAGTCTGTGTGATCATGGCTTT |
| *Zm00001d039645* | CACCTTCTTCCTCCTCAC | GTAGTCGTCGATGGAGAG |
| *CULLIN2* | GAAGAGCCGCAAAGTTATGG | ATGGTAGAAGTGGACGCACC |

^a^Candidate genes of *qRPR3-1* and reference gene
