# Supplementary material for: Characterization and Fine Mapping of qRPR1-3 and qRPR3-1, Two Major QTLs for Rind Penetrometer Resistance in Maize
Source: Front Plant Sci. 2022 Jul 19;13:944539. doi: 10.3389/fpls.2022.944539 (PMC9344970; doi:10.3389/fpls.2022.944539)
Supplement: Supplementary file 2 [file Image_1.PDF]

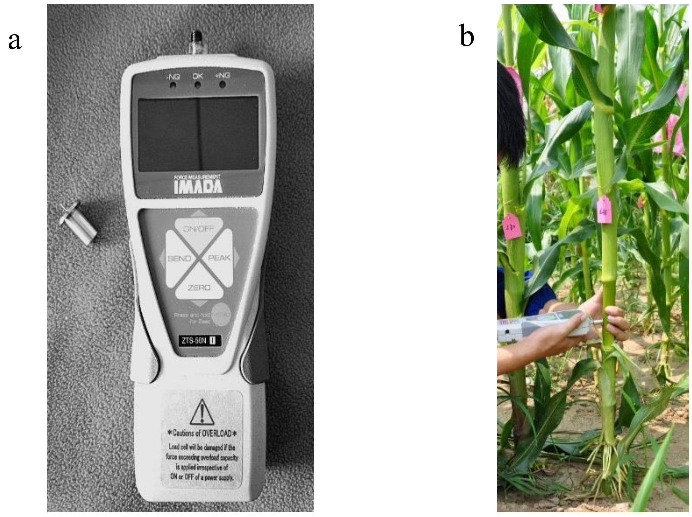

Supplemental Fig. S1. The detecting machine of stalk stiffness. (a) IMADA digital force gauge for testing stalk rind penetrometer resistance (RPR). (b) The measurement of RPR was performed at the third internodes below the primary ear.

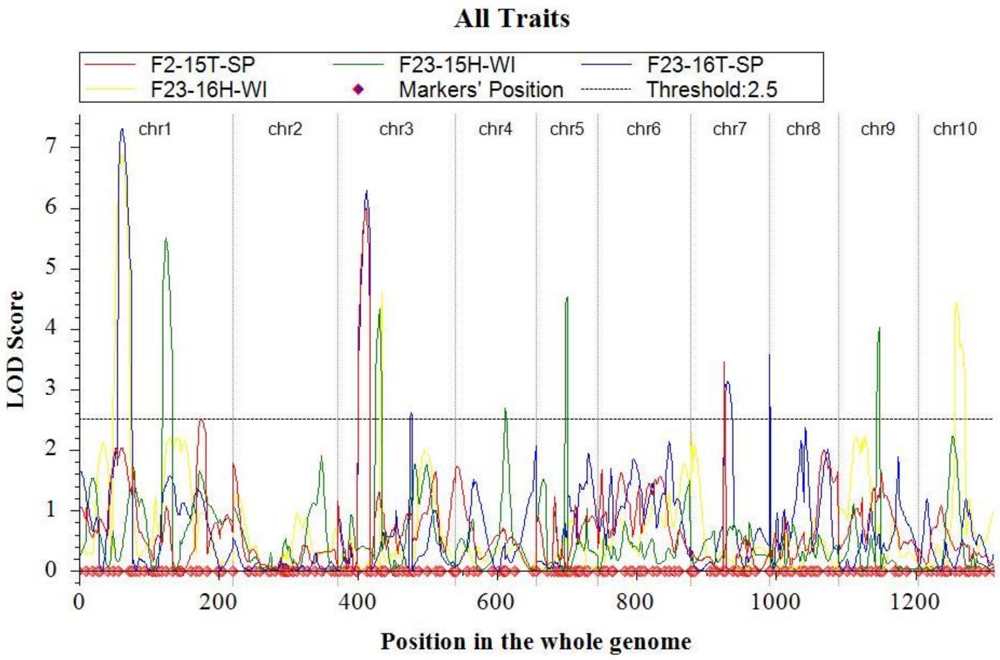

Supplemental Fig. S2. Genome-wide logarithm of odds (LOD) profile of quantitative trait loci (QTL) for RPR in F_2 and $F_{2:3}$ populations. T: Tai'an, H: Hainan, SP: spring, WI: winter.
